# A Comparative MRI Analysis of a Juvenile Non-Human Primate and a Paediatric Human Brain: A Guide for Malaysian Neuroscientists

**DOI:** 10.21315/mjms-05-2026-297

**Published:** 2026-06-30

**Authors:** Wan Nor Adibah Wan Zakaria, Nur Asma Sapiai, Siti Fatimah Mukhtar, Mohd Zulkifli Mustafa, Farizan Ahmad, Zaitun Zakaria, Anani Aila Mat Zin, Jafri Malin Abdullah

**Affiliations:** 1 Department of Neurosciences, School of Medical Sciences, Health Campus, Universiti Sains Malaysia, 16150 Kubang Kerian, Kelantan, Malaysia; 2 Department of Radiology, School of Medical Sciences, Health Campus, Universiti Sains Malaysia, 16150 Kubang Kerian, Kelantan, Malaysia; 3 Brain and Behaviour Cluster, Hospital Pakar Universiti Sains Malaysia, Universiti Sains Malaysia, Health Campus, Jalan Raja Perempuan Zainab 2, 16150 Kota Bharu, Kelantan, Malaysia; 4 Department of Anatomy, School of Medical Sciences, Health Campus, Universiti Sains Malaysia, 16150 Kubang Kerian, Kelantan, Malaysia; 5 Department of Pathology, School of Medical Sciences, Health Campus, Universiti Sains Malaysia, 16150 Kubang Kerian, Kelantan, Malaysia

**Keywords:** non-human primate, human, brain, neuroanatomy, Macaca nemestrina

## Abstract

**Background:**

Comparative neuroimaging of non-human primates (NHPs) and humans provides crucial insights into evolutionary biology and translational neuroscience. However, accessible, metric-driven structural guides tailored for emerging neuroimaging communities, such as in Malaysia, remain limited. This study presents a comparative magnetic resonance imaging (MRI) analysis of a juvenile NHP and a paediatric human brain to establish a foundational structural reference guide.

**Methods:**

T1- and T2-weighted MRI sequences were evaluated for tissue contrast consistency. Macrostructural and microstructural metrics, including craniofacial dimensions, skull base angles, corpus callosum thickness, and deep grey matter volumes, were quantified. Inter-rater reliability was assessed using the intraclass correlation coefficient (ICC), and independent *t*-tests evaluated cross-species variations.

**Results:**

Both species exhibited highly conserved tissue contrast patterns, characterised by grey matter hypointensity on T1-weighted images and hyperintensity on T2-weighted sequences. However, significant structural divergence was identified in specialised regions; the human brain demonstrated marked cranial base flexion (133° vs. 149° in the NHP) in addition to a profoundly expanded corpus callosum (genu: 1.05 cm vs. 0.28 cm) and a pronounced, non-uniform enlargement of higher-order frontal and temporal association cortices.

**Conclusion:**

While fundamental tissue and structural architectures are highly conserved, the human paediatric brain displays selective, non-uniform expansion of higher-order cognitive networks rather than simple scaling. This comparative baseline validates the translational utility of NHP models while providing a practical, localised anatomical guide for Malaysian neuroscientists.

## Introduction

Comparative evaluation of brain magnetic resonance imaging (MRI) in non-human primates (NHPs) and humans demonstrates substantial structural concordance alongside clear anatomical distinctions, reflecting evolutionary specialisation of the human brain. Despite differences in brain size and organisational complexity, fundamental MRI tissue contrast characteristics are preserved across species, enabling meaningful cross-species structural comparisons (1–3). These similarities have supported the widespread use of macaque models in structural and functional neuroimaging research, particularly in studies investigating cortical organisation, subcortical circuitry, and brain connectivity (1, 3, 4).

Advances in high-resolution MRI atlases and population-based templates have further improved the accuracy of macaque brain mapping and cross-species comparisons, allowing detailed structural characterisation of cortical and subcortical regions (4–6). These developments have strengthened the role of NHP as translational models in neuroscience and neuroimaging research.

At the same time, human brain development is characterised by progressive cortical expansion, increasing gyrification, and maturation of white matter pathways during early life, which can be visualised using paediatric MRI (7–9). Comparative neuroimaging, therefore, provides a valuable framework for understanding both conserved neuroanatomical architecture and species-specific evolutionary adaptations.

The objective of this study is to evaluate the extent to which non-human primate brain MRI recapitulates human neuroanatomical features, thereby informing the translational applicability of primate models in neuroimaging research (1, 3, 4).

## Methods

### Ethical Approval

The animal study was conducted following ethical approval from the Universiti Sains Malaysia Institutional Animal Care and Use Committee (USM IACUC), certificate USM/ANIMAL ETHICS APPROVAL/2015/(95) {648}, dated 4 May 2021, valid until 1 August 2022, as well as receipt of an Approval Permit from the Headquarters, Department of Wildlife and National Parks Peninsula Malaysia: JPHL&TN(IP):100-34/1.24 Jld 17(39), dated 13 April 2021, valid until 1 August 2022, Approval number: BPKS(8.15)248-11(29), receipt number 100315000017. Human data were retrieved under ethical approval to store the neuroimaging data by the Human Research Ethics Committee (JEPeM) of Universiti Sains Malaysia (USM/JEPeM/HHL/2023/02).

### Study Subject

An 18-month-old female Macaca nemestrina (pig-tailed macaque), designated “Bulma,” was used as the animal subject. For human comparison, a seven-year-old female child was selected after age calculation was done based on https://tohumanyears.com/info/monkey-years-to-human-years/. The human subject was selected using purposive sampling to match the required demographic profile, successfully fulfilling all age, sex, and normative structural MRI criteria.

The human paediatric MRI data were selected based on strict normative criteria, including the absence of known neurological disorders, developmental delays, or trauma. The scan was formally reviewed and cleared of gross structural abnormalities by a neuroradiologist.

Similarly, the juvenile NHP subject was under continuous veterinary care, displaying age-appropriate development with no history of neurological or systemic illness. Gross visual inspection of both T1-weighted and T2-weighted structural MR images confirmed the absence of focal lesions, mass effects, or significant artefacts, rendering both subjects suitable as normal anatomical baselines.

### MRI Acquisition Protocol

Magnetic resonance imaging of both subjects was conducted using a 3.0-Tesla Philips Achieva Best, the Netherlands, system in the MRI suite of the Radiology Department, Hospital Pakar Universiti Sains Malaysia (HPUSM). Total imaging time ranged from 30 to 45 minutes. Subjects were positioned supine on the MRI gantry and fitted with a standard head coil (SENSE Head 8-channel). Head stabilisation was achieved using foam padding to minimise motion artefacts during image acquisition.

Detailed MRI acquisition parameters for both the human and animal subjects are summarised in Table 1.

Image post-processing and quantitative analyses were performed at the MRI Suite, Radiology Department, HPUSM, by a neuroscientist and a neuroradiologist using the dedicated image analysis software Philips Intellispace Portal Software version 12.1.

All measurements were obtained in triplicate to ensure technical reproducibility. Independent assessments were conducted by a second rater, and inter-rater reliability for continuous measurements was evaluated using the intraclass correlation coefficient (ICC) to assess agreement between raters. Independent *t*-tests were conducted using Microsoft Excel to assess differences in continuous measurement values between the two groups.

The superior frontal gyrus, superior temporal gyrus, and hippocampus were measured in coronal oblique view at the level of the foramen of Monro. The precentral gyrus and postcentral gyrus were measured in axial view, while the occipital lobe was measured in sagittal view at the parasagittal level.

## Results

On the T1-weighted sequences, both the NHP and human brain demonstrated consistent contrast differentiation, with grey matter appearing relatively hypointense and white matter hyperintense. This pattern is reversed on the T2-weighted sequences, where grey matter appears hyperintense and white matter hypointense. These conserved signal characteristics across species reflect similarities in tissue composition and water distribution, thereby supporting meaningful comparative interpretation (Figures 1 and 2).

The structural comparisons demonstrate marked proportional differences that are functionally and evolutionarily significant. The NHP brain is notably smaller in total volume, with reduced proportional size across the cortical and subcortical regions (Table 2). For instance, the eye globes in the NHP (Figure 3A) measure approximately 1.8 cm in the anteroposterior (AP) axis, compared to 2.3 cm in the human (Figure 3B). This reflects both the smaller craniofacial dimensions of the NHP and the variations in orbital orientation and skull base structure. Notably, the skull base angle differs markedly: 149° in the NHP versus 133° in the human (Figure 4). This difference reflects the evolutionary transition from a horizontal cranial base in quadrupedal primates to a more flexed cranial base in bipedal humans, accommodating upright posture, a forward-shifted foramen magnum, and expanded frontal lobe volume.

The mid-sagittal T1-weighted MR images provide a clear view of conserved and divergent features along the brain’s longitudinal axis. The corpus callosum is present in both species, confirming interhemispheric connectivity, but shows profound size differences. In the NHP [Figure 4(A)], the genu and splenium measure 0.28 cm and 0.23 cm, respectively, while in the human [Figure 4(B)], these structures measure 1.05 cm and 0.86 cm, respectively. This significant increase in humans supports greater interhemispheric communication and higher-order integrative functions, reflecting the demands of complex cognition, language, and sensorimotor integration. While the corpus callosum is consistently visualised in the NHP, its reduced size may also be species-specific and must be interpreted with caution across different primate taxa.

Deep grey matter structures show consistent topology but reduced volume in the NHP brain. The lentiform nuclei in the NHP appear smaller and less conspicuous (Figure 5), measuring 1.71 cm in AP extent, while being larger and more defined in the human, namely 3.84 cm. Similarly, the thalamus and head of the caudate nucleus are substantially larger in humans. These subcortical structures are integral to sensorimotor regulation and cognitive processing, and their enlargement in humans correlates with increased neurofunctional demands and the need for more complex circuitry.

The pituitary gland also demonstrates proportional differences, measuring approximately 0.24 cm in height in the NHP and 0.46 cm in the human. The larger human pituitary reflects the expanded role of the hypothalamic–pituitary axis in endocrine regulation and systemic homeostasis. The posterior pituitary bright spot, though present in both, is more prominent in the human, further affirming similarities in neurosecretory function and imaging characteristics and offering a useful reference for evaluating pituitary disorders in comparative settings. The posterior pituitary bright spot, typically seen on T1-weighted images due to the presence of neurosecretory granules, is present in both species but appears relatively larger and more intense in the human, indicating consistency in neurohypophysis function across primates.

The measurement of the area of gyrus in the human also shows differentiation of values compared to the NHP (Table 3, Figures 6 and 7). Compared to monkeys, the human brain shows disproportionate enlargement of higher-order association areas, particularly the frontal and temporal lobes.

Independent *t*-tests between the two data sets were conducted as two-sided tests, with statistical significance defined as *P* < 0.01. Inter-rater reliability for continuous measurements was assessed using the ICC based on a two-way mixed-effects model with absolute agreement. The ICC demonstrated excellent reliability (ICC ≈ 0.95), indicating near-perfect agreement between raters. In view of the exploratory study design and limited sample size, all findings were interpreted with appropriate caution.

## Discussion

The present comparative MRI analysis demonstrates that several fundamental neuroimaging characteristics are conserved between NHPs and humans. On both the T1- weighted and T2-weighted sequences, similar tissue contrast patterns were observed, with grey matter appearing relatively hypointense compared with white matter on T1-weighted imaging and becoming hyperintense on T2- weighted sequences. These signal characteristics reflect intrinsic differences in tissue composition, including myelin content, cellular density, and water distribution, which remain broadly consistent across primate species (1–3, 6). The preservation of these signal patterns supports the use of NHP models for comparative neuroimaging studies and enables reliable cross-species interpretation of structural brain anatomy.

Despite these shared imaging characteristics, clear structural differences are evident. The NHP brain is substantially smaller in overall volume compared with the human brain, reflecting evolutionary divergence in cortical expansion and cranial development. This difference is evident not only in global brain dimensions but also in associated craniofacial structures. For example, the anteroposterior dimension of the ocular globes in the NHP measures approximately 1.8 cm compared with 2.3 cm in humans. These differences correspond to the smaller craniofacial skeleton and altered orbital configuration in NHPs. Furthermore, the skull base angle differs considerably between the species, measuring approximately 149° in NHPs compared with 133° in humans. This anatomical variation reflects the evolutionary transition from the relatively horizontal cranial base of quadrupedal primates to the more flexed cranial base seen in bipedal humans, which accommodates anterior displacement of the foramen magnum and expansion of the frontal lobes associated with higher cognitive function (10).

Mid-sagittal MRI images further highlight both conserved and divergent neuroanatomical features. The corpus callosum is clearly identifiable in both species, confirming the presence of interhemispheric connectivity within the primate brain. However, marked proportional differences are observed. In the NHP brain, the genu and splenium measure approximately 0.28 cm and 0.23 cm, respectively, whereas in the human brain these structures measure approximately 1.05 cm and 0.86 cm, respectively. This substantial enlargement in humans reflects increased interhemispheric communication required for complex integrative processes, including language, executive function, and advanced sensorimotor co-ordination. While the corpus callosum is consistently visualised in the NHP, its reduced size may reflect both species-specific neuroanatomical scaling and functional specialisation (1, 3, 5, 10).

Deep grey matter structures demonstrate similar spatial organisation but differ in absolute size and prominence. The basal ganglia, including the lentiform nuclei, appear smaller and less conspicuous in the NHP brain compared with humans. In the present observations, the anteroposterior dimension of the lentiform nucleus in the NHP measures approximately 1.7 cm, while the basal ganglia structures in humans demonstrate larger dimensions and clearer structural definition. Likewise, the thalamus and the head of the caudate nucleus are significantly larger in humans. These nuclei are essential components of cortico-subcortical circuits responsible for motor regulation, cognitive integration, and behavioural modulation. Their relative enlargement in humans likely reflects the increased complexity of neural networks supporting higher cognitive functions (1, 4, 6).

The pituitary gland also demonstrates measurable interspecies differences. In the NHP brain, the pituitary gland measures approximately 0.24 cm in height compared with approximately 0.46 cm in humans. This proportional enlargement in humans may reflect the greater complexity of the hypothalamic–pituitary axis in regulating endocrine and metabolic functions. Despite these differences in size, the posterior pituitary bright spot is visible in both species on T1-weighted imaging. This hyperintense signal, attributed to the presence of neurosecretory granules containing vasopressin and oxytocin, indicates preserved neuro-hypophyseal function across primate species. Interestingly, the bright spot appears more prominent in humans, which may reflect differences in glandular volume or hormonal storage capacity. The consistent presence of this imaging feature across species further reinforces the biological similarity of hypothalamic–pituitary mechanisms (7).

Advances in neuroimaging technology have further improved the ability to perform detailed cross-species comparisons of brain anatomy. Modern MRI-based atlases and digital brain templates have enabled high-resolution mapping of cortical and subcortical structures in macaques, facilitating alignment of experimental imaging data with standardised stereotaxic co-ordinates (2–4, 6). These atlases provide valuable frameworks for studying functional connectivity, structural organisation, and translational neuroscience applications in NHP models.

In addition, developmental neuroimaging studies in humans have demonstrated progressive maturation of cortical and subcortical structures during early life, including increasing cortical folding and white matter myelination (8, 9). Understanding these developmental processes is important when interpreting comparative brain morphology, particularly when evaluating younger primate specimens or juvenile animal models. To establish a valid, translationally matched baseline between our younger subjects, we cross-referenced the primate developmental and chronological lifespans with human trajectories using the foundational lifetable profiles developed by Tigges et al. (11). Their survivorship paradigms confirm that developmental milestones between juvenile macaques and paediatric human cohorts scale predictably, thereby justifying this structural comparison and minimising maturity as an unaddressed confounding variable (11).

The data demonstrate that the human brain is not just a scaled-up version of the monkey brain but shows selective and disproportionate expansion of key cortical regions. The frontal and temporal lobes are markedly enlarged, supporting higher-order cognition, executive function, and language. This selective cortical regionalisation mirrors the in vivo structural findings of Croxson et al. (12), who demonstrated that while primary sensory and motor networks remain highly conserved across primate species, phylogenetically recent heteromodal association cortices exhibit massive evolutionary expansion alongside high structural variability in humans. Conversely, the hippocampus and sensorimotor areas show more moderate, proportional increases, reflecting preserved but enhanced memory and motor functions. The occipital lobe exhibits a large absolute increase, indicating strong visual processing in both species, though not uniquely specialised for humans. Compared to monkeys, human brain evolution reflects selective expansion of association cortices rather than uniform growth, underpinning uniquely advanced cognitive and linguistic abilities (12).

Collectively, these findings demonstrate that while the NHP brain shares the fundamental structural organisation of the human brain, substantial proportional differences exist across cortical, subcortical, and cranial structures. Classical stereotaxic atlases remain important references for understanding macaque brain organisation and continue to guide comparative neuroanatomical research (13). Nevertheless, the preservation of overall neuroanatomical architecture supports the continued use of NHPs as valuable translational models for neuroimaging research. Comparative MRI analysis, therefore, provides important insights into both the evolutionary development of the primate brain and the applicability of NHP models in neuroscience and clinical neuroradiology.

However, several limitations must be considered when extrapolating findings from NHP models to human neuroscience. The differences in cortical gyrification, neocortical expansion, and interhemispheric connectivity reflect evolutionary adaptations related to higher-order cognition in humans. As a result, while macaques provide excellent models for studying fundamental neurobiological processes, they may be less suitable for investigating disorders primarily involving complex cognitive networks, such as schizophrenia, autism spectrum disorders, and advanced language processing.

From a clinical imaging perspective, understanding species-specific anatomical variation is crucial for accurate interpretation of comparative MRI findings. Recognition of normal proportional differences in cortical thickness, ventricular size, and commissural structures helps prevent misinterpretation of normal anatomical variants as pathological abnormalities. Moreover, awareness of differences in cranial base orientation and skull architecture may assist in interpreting structural brain alignment when translating experimental imaging protocols from animal models to human clinical applications.

Overall, comparative MRI studies between macaques and humans contribute significantly to our understanding of brain evolution and neuroanatomical organisation. By integrating classical anatomical knowledge with modern neuroimaging techniques, such studies strengthen the translational bridge between experimental neuroscience and clinical neuroradiology. The continued development of advanced primate brain atlases and imaging methodologies will further enhance the utility of NHP models in neuroscience research and support more accurate interpretation of cross-species neuroimaging data.

## Conclusion

This comparative MRI analysis demonstrates that, despite differences in brain size and cortical complexity, monkey and human brains share conserved structural organisation and MRI signal characteristics. Core features such as grey–white matter contrast, deep grey matter topology, brainstem anatomy, ventricular configuration, and pituitary imaging are preserved across species, supporting the use of NHPs as translational models in neuroimaging research. However, notable differences in cortical gyrification, corpus callosum size, skull base angle, and overall brain volume reflect evolutionary adaptations linked to higher cognitive function in humans. These findings highlight the need for careful interpretation when translating non-human primate data to human neuroscience and emphasise the value of comparative MRI in understanding primate brain organisation.

## Figures and Tables

**Figure 1:**
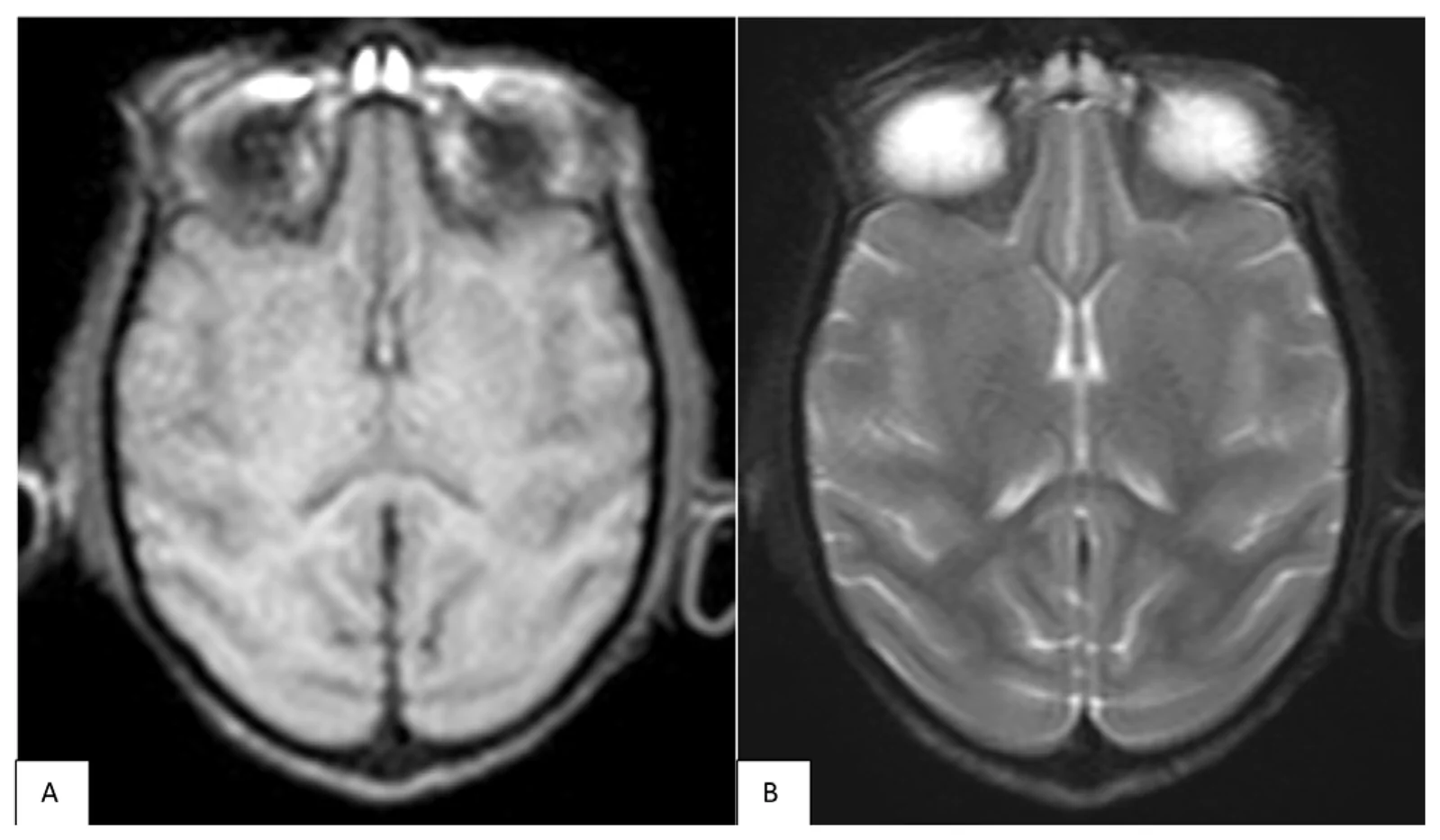
Axial T1WI (A) and axial T2WI(B) of NHP at level of basal ganglia

**Figure 2:**
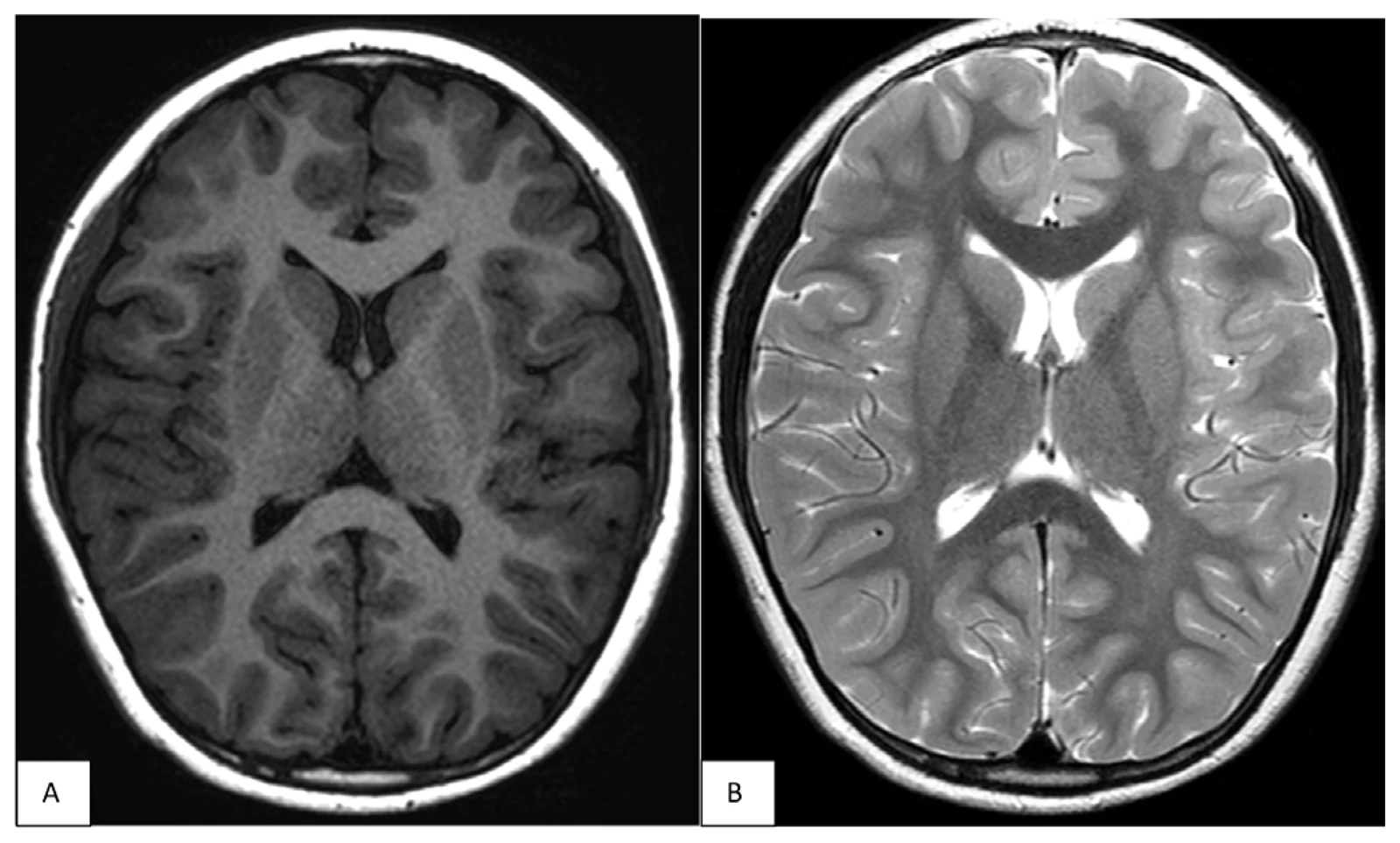
Axial T1WI (A) and axial T2WI(B) of human at level of basal ganglia

**Figure 3:**
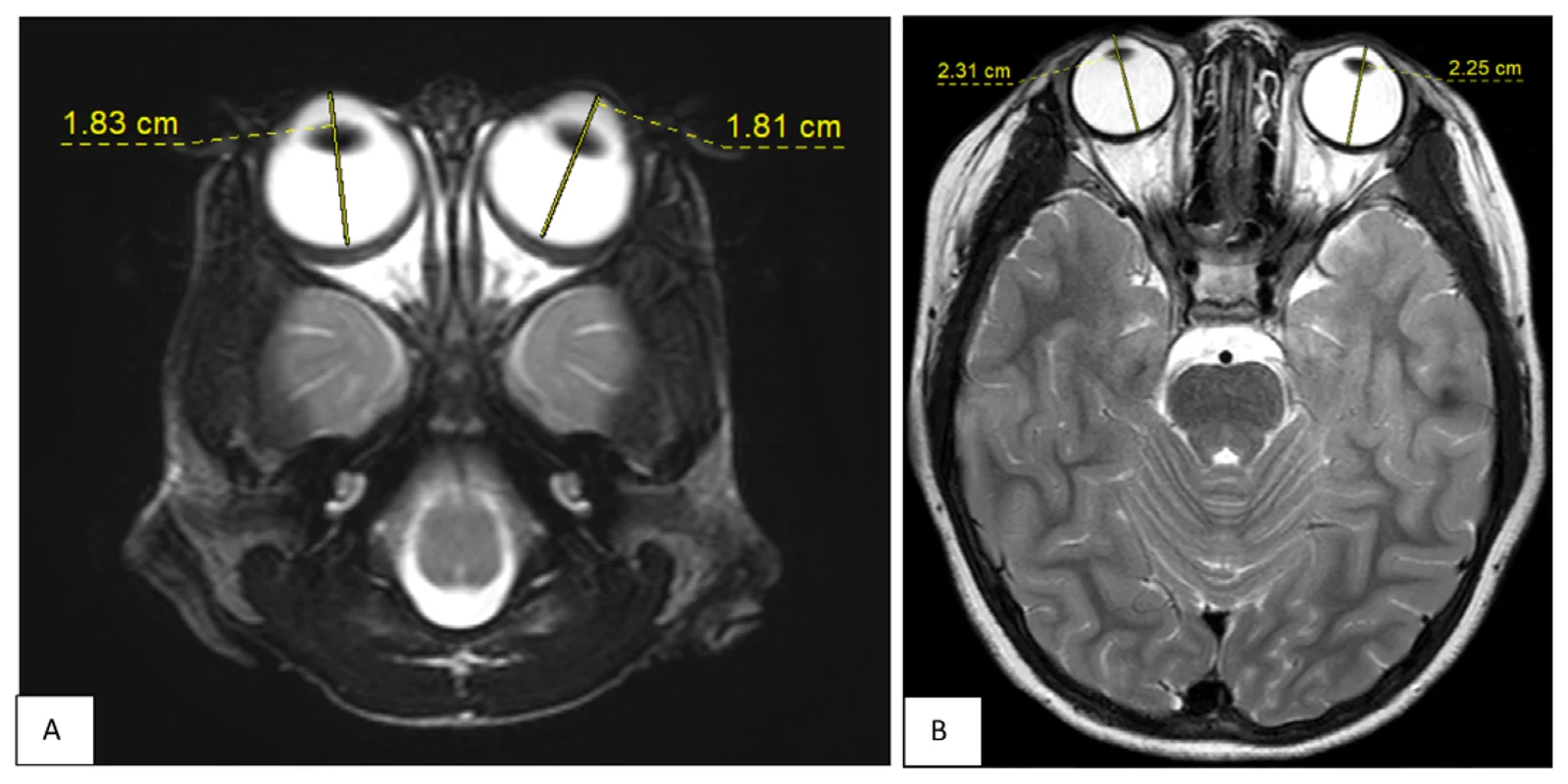
The difference in size of eye globes of NHP (A) and human (B)

**Figure 4:**
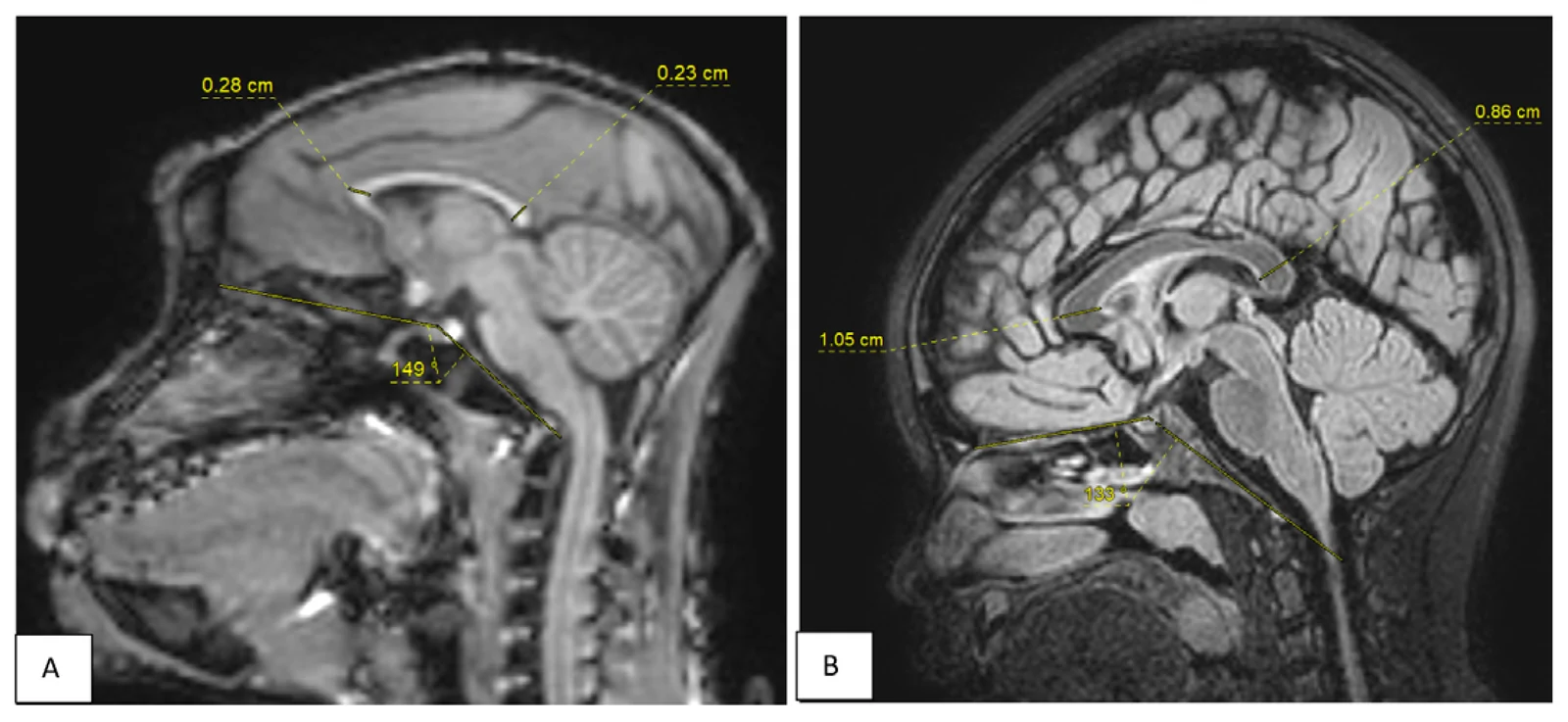
Sagittal T1 of NHP (A) and Sagittal T1 of human (B)

**Figure 5:**
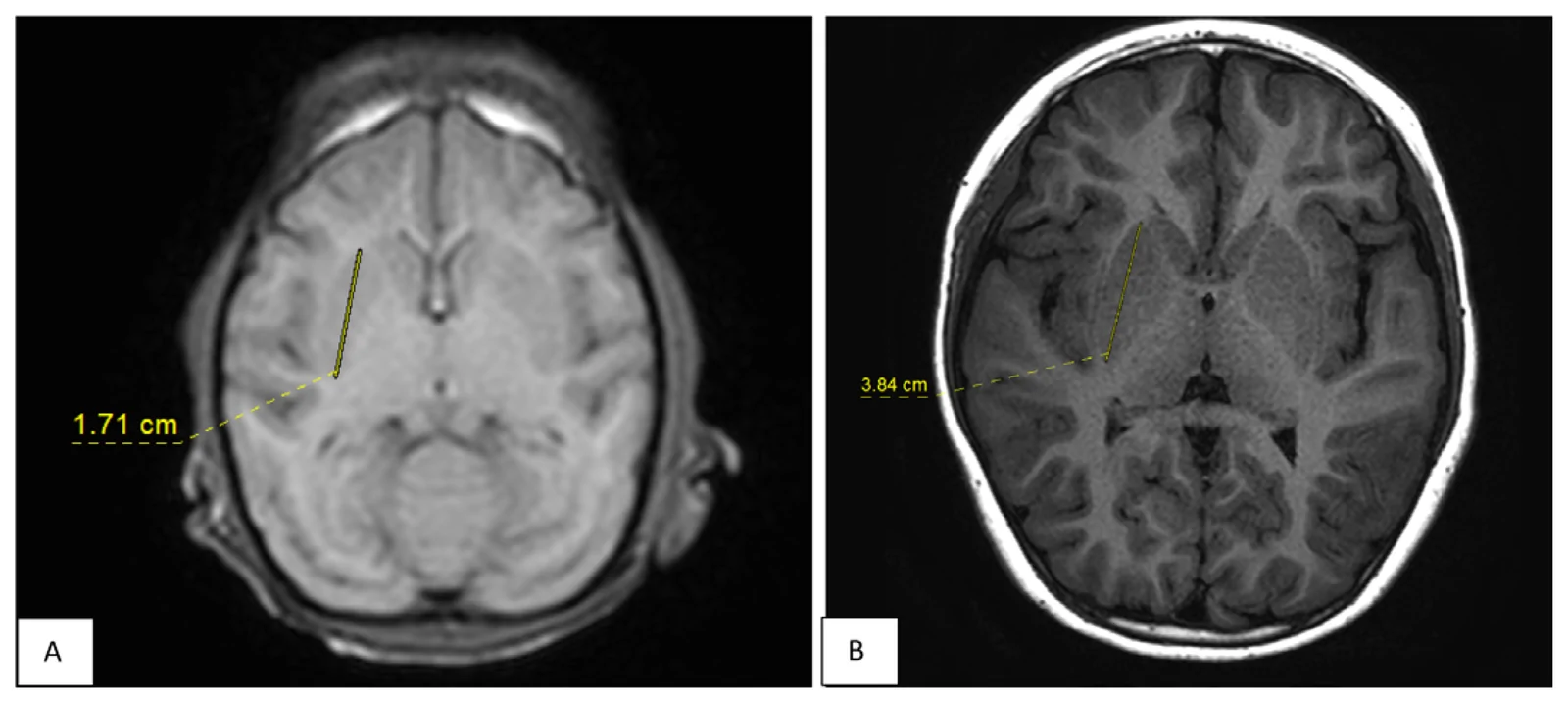
Axial T1 of NHP (A) and human (B) at level lentiform nuclei

**Figure 6:**
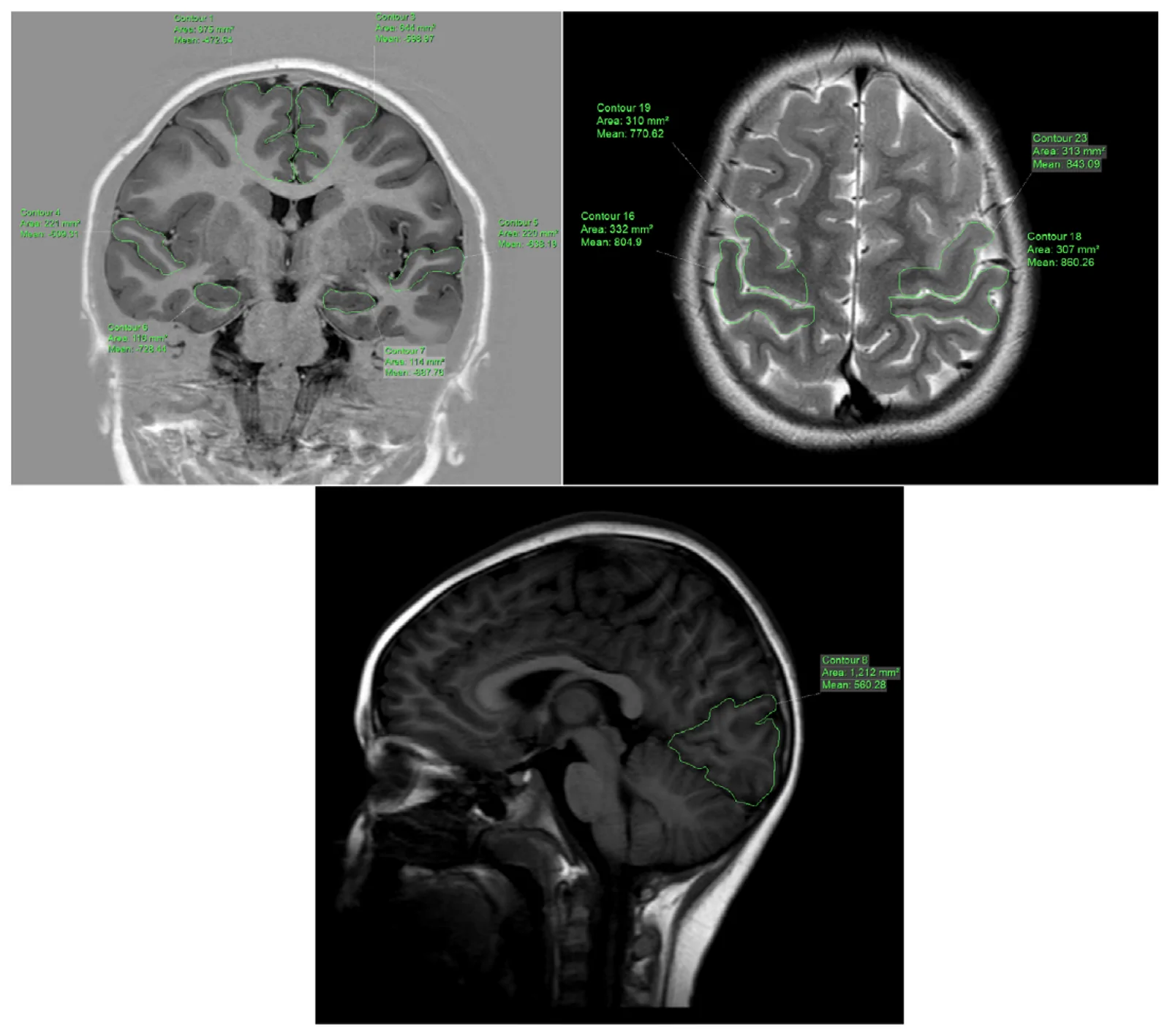
Measurement of the bilateral gyrus and the left occipital lobe of human

**Figure 7:**
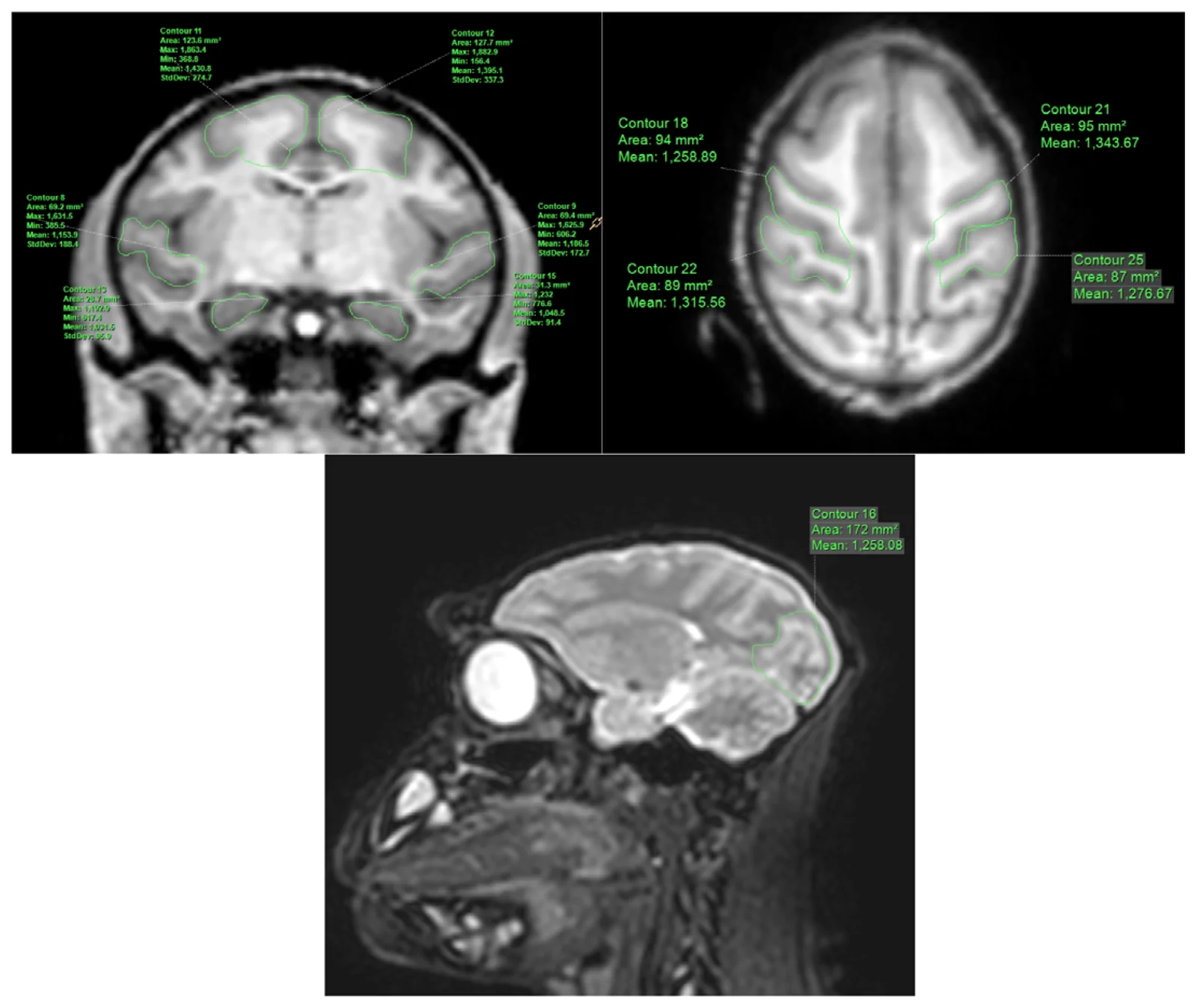
Measurement of the bilateral gyrus and the left occipital lobe of NHP

**Table 1:** MRI acquisition parameters for human and NHP.

Sequence	Slice thickness (mm)	FOV (mm)	TR / TE / TI (ms)	No. of slices	Spatial resolution (mm)/ Voxel	Matrix	Gap	Scan duration (min)
**Female human**								
Saggital T1-weighted IR	5	230 × 261 × 132	2,000 / 15 / 800	24	0.85 × 1.13	272 × 230	0.5	2.18
Axial T1-weighted IR	3	210 × 210 × 147	2,000 / 33 / 800	37	0.91 × 0.91	480 × 480	1	5.52
Axial T2 - weighted	3	220 × 177 × 147	4,000 / 97	37	0.57 × 0.73	512 × 512	1	2.48
3D Sagital FLAIR	1.11	250 × 250 × 180	4,800 / 340 / 1,650	321	1.12 × 1.12	240 × 240	0.6	4.48
**NHP**								
Saggital T1- weighted	3	131 × 131	8.8 / 4.1	23	0.85 × 1.13	512 × 512	0.5	2.18
Axial T1-weighted IR	3	150 × 150	2,000 / 33	20	0.91 × 0.91	336 × 336	1	3.00
Axial T2 - weighted	3	150 × 150	2,325.1 / 110	20	0.57 × 0.73	320 × 320	1	1.50
3D Saggital FLAIR	1.1	200 × 200	4,800 / 300 / 1650	180	1.12 × 1.12	224 × 224	0.6	4.48

FOV = field of view; TR = repetition time; TE = echo time; TI = inversion time; NHP = non-human primate

**Table 2:** Comparing the anatomy measurements of specific regions between NHP and human

Anatomy	NHP (cm)	Human (cm)		*P*-value
Eye globes (anteroposterior diameter)	1.80	2.30	0.0316	*P* < 0.05
Genu of corpus callosum	0.28	1.05	0.0023	*P* < 0.005
Splenium of corpus callosum	0.23	0.86	2.40969E-05	*P* < 0.005
Lentiform nuclei	1.71	3.84	0.0001	*P* < 0.001
Pituitary gland	0.24	0.46	0.0215	*P* < 0.05
Skull base angle (°)	149.00	133.00	0.0026	*P* < 0.005

**Table 3:** Comparing the area of specific regions between NHP and human

Area	NHP (mm^2^)	Human (mm^2^)	*P*-value	
Right superior frontal gyrus	123.6	675	3.32106E-34	*P* < 0.0001
Left superior frontal gyrus	127.7	644	0.00000988	*P* < 0.0001
Right superior temporal gyrus	69.2	221	0.00000281	*P* < 0.0001
Left superior temporal gyrus	69.4	220	0.000000658	*P* < 0.0001
Right hippocampus	28.7	116	0.0000267	*P* < 0.0001
Left hippocampus	31.3	114	0.000000471	*P* < 0.0001
Right occipital lobe	168	1181	0.000000000177	*P* < 0.0001
Left occipital lobe	172	1,212	0.00000000586	*P* < 0.0001
Right postcentral gyrus	89	332	0.000000431	*P* < 0.0001
Left postcentral gyrus	87	307	0.000000571	*P* < 0.0001
Right precentral gyrus	94	310	0.000000603	*P* < 0.0001
Left precentral gyrus	95	313	0.00000000118	*P* < 0.0001

## Data Availability

The data that support the findings of this study are available from the corresponding author upon reasonable request. Due to institutional and ethical restrictions, the data are not publicly available as they contain information that could compromise patient privacy.

## References

[1] Saleem KS, Logothetis NK (2006). A combined MRI and histology atlas of the rhesus monkey brain in stereotaxic coordinates.

[2] McLaren DG, Kosmatka KJ, Oakes TR, Kroenke CD, Kohama SG, Matochik JA (2009). A population-average MRI-based atlas collection of the rhesus macaque. Neuroimage.

[3] Frey S, Pandya DN, Chakravarty MM, Bailey L, Petrides M, Collins DL (2011). An MRI-based average macaque monkey stereotaxic atlas and space (MNI monkey space). Neuroimage.

[4] Lu Y, Cui Y, Cao L, Dong Z, Cheng L, Wu W (2024). Macaque brainnetome atlas: a multifaceted brain map with parcellation, connection, and histology. Sci Bull.

[5] Moirano JM, Bezgin GY, Ahlers EO, Kötter R, Converse AK (2019). Rhesus macaque brain atlas regions aligned to an MRI template. Neuroinformatics.

[6] Reveley C, Gruslys A, Ye FQ, Glen D, Samaha J, Russ BE (2017). Three-dimensional digital template atlas of the macaque brain. Cereb Cortex.

[7] Barkovich AJ, Raybaud C (2019). Pediatric neuroimaging.

[8] Knickmeyer RC, Gouttard S, Kang C, Evans D, Wilber K, Smith JK (2008). A structural MRI study of human brain development from birth to 2 years. J Neurosci.

[9] Shi F, Yap PT, Wu G, Jia H, Gilmore JH, Lin W (2011). Infant brain atlases from neonates to 1- and 2-year-olds. PLoS One.

[10] LeMay M (1976). Morphological cerebral asymmetries of modern man, fossil man, and nonhuman primate. Ann N Y Acad Sci.

[11] Tigges J, Gordon TP, McClure HM, Hall EC, Peters A (1988). Survival rate and life span of rhesus monkeys at the Yerkes regional primate research center. Am J Primatol.

[12] Croxson PL, Forkel SJ, Cerliani L, Thiebaut de Schotten M (2017). Structural variability across the primate brain: a cross-species comparison. Cereb Cortex.

[13] Snider RS, Lee JC (1961). A stereotaxic atlas of the monkey brain (Macaca mulatta).

